# ADAM-1: An AI Reasoning and Bioinformatics Model for Alzheimer’s Disease Detection and Microbiome-Clinical Data Integration

**DOI:** 10.1109/access.2025.3599857

**Published:** 2025-08-18

**Authors:** ZIYUAN HUANG, VISHALDEEP KAUR SEKHON, ROOZBEH SADEGHIAN, MARIA L. VAIDA, CYNTHIA JO, BETH A. MCCORMICK, DOYLE V. WARD, VANNI BUCCI, JOHN P. HARAN

**Affiliations:** 1 Department of Microbiology, UMass Chan Medical School, Worcester, MA 01655, USA; 2 Department of Emergency Medicine, UMass Chan Medical School, Worcester, MA 01655, USA; 3 Program in Microbiome Dynamics, UMass Chan Medical School, Worcester, MA 01655, USA; 4 Department of Geriatric Medicine and Gerontology, Johns Hopkins University, Baltimore, MD 21224, USA; 5 School of Analytics and Computational Sciences, Program in Data Sciences, Harrisburg University of Science and Technology, Harrisburg, PA 17101, USA

**Keywords:** Alzheimer’s disease, artificial intelligence, multi-agent systems, knowledge based systems

## Abstract

Alzheimer’s Disease Analysis Model Generation 1 (ADAM-1) is a multi-agent reasoning large language model (LLM) framework designed to integrate and analyze multimodal data, including microbiome profiles, clinical datasets, and external knowledge bases, to enhance the understanding and classification of Alzheimer’s disease (AD). By leveraging the agentic system with LLM, ADAM-1 produces insights from diverse data sources and contextualizes the findings with literature-driven evidence. A comparative evaluation with XGBoost revealed a significantly improved mean F1 score and significantly reduced variance for ADAM-1, highlighting its robustness and consistency, particularly when utilizing human biological data. Although currently tailored for binary classification tasks with two data modalities, future iterations will aim to incorporate additional data types, such as neuroimaging and peripheral biomarkers, and expand them to predict disease progression, thereby broadening ADAM-1’s scalability and applicability in AD research and diagnostic applications.

## I. INTRODUCTION

The integration of multimodal data sources in biomedical research has accelerated with advances in deep learning, particularly through the development of large language models (LLMs). The introduction of AlexNet in 2012 demonstrated the potential of deep neural networks for complex tasks such as image classification [1]. Subsequent developments, including transformer architecture in 2017 and the release of models like GPT-1 in 2018 and GPT-2 in 2019, marked significant milestones in natural language processing [2], [3]. Other transformer-based LLM families, such as LLaMA, Gemini, Claude, and DeepSeek, have also demonstrated powerful structured and unstructured biomedical data processing [4], [5], [6], [7], [8], [9], [10]. More recently, reasoning-focused LLMs, including GPT-4.5, OpenAI o1, Gemini 2.5, Claude Sonnet 3.7, and DeepSeek R1, have further extended the role of artificial intelligence (AI) in scientific domains by enabling more complex inference and task coordination [11], [12], [13], [14], [15].

In bioscience, LLMs have shown promise in clinical decision support, patient-trial matching, and biomedical question answering [16], [17], [18]. Concurrent developments like AlphaFold and ESM-2 have led to breakthroughs in protein structure prediction and variant interpretation [19], [20]. Frameworks like BioLunar, ASD-cancer, and CHIEF exemplify the value of integrating multimodal evidence, including genomic, imaging, and text-based modalities, for enhanced diagnostics in cancer and other diseases [21], [22], [23].

While cancer research has rapidly integrated AI tools across diagnostics, treatment planning, and drug discovery, research into Alzheimer’s disease (AD) has been slower to adopt these technologies on a comparable scale. AD is a multifactorial neurodegenerative disorder characterized by beta-amyloid plaques, tau protein tangles, immune system dysregulation, and changes in the gut microbiome [24], [25]. Traditional studies often depend on single-modality datasets, which can limit comprehensive insights into the disease’s progression.

This study presents the Alzheimer’s Disease Analysis Model Generation 1 (ADAM-1), a multi-agent reasoning framework leveraging large language models (LLMs) and retrieval-augmented generation (RAG) [26]. The current version, Generation 1 (ADAM-1, hereafter referred to as ADAM), integrates clinical and microbiome data, modular agents with Chain-of-Thought (CoT) [27] reasoning to enhance interpretability and contextual relevance in disease classification. Trained and tested on a laboratory dataset of 335 multimodal data samples from older adults, ADAM achieved a significantly higher mean F1 score and a much lower prediction variance than the XGBoost baseline model. These findings highlight the methodological benefits of agentic reasoning systems in maintaining performance under data-limited situations.

## II. MULTIMODAL DATASET DESCRIPTION AND VISUALIZATION

This study utilizes the nursing home clinical and metagenomic dataset originally compiled by Haran et al. [28], which focused on the dysregulation of the anti-inflammatory P-glycoprotein pathway in AD, thereby providing valuable insights into microbiome-host interactions that may underlie AD pathogenesis and inform future therapeutic strategies. The original study received approval from the Institutional Review Board (IRB) of the University of Massachusetts Medical School (Docket H00010892), and informed consent was obtained from all participants. For the present analysis, only de-identified data were used. We repurposed this dataset to develop ADAM, an LLM-based classifier and reporting system. The dataset comprises clinical and microbiome data from five nursing home sites in central Massachusetts, focusing on individuals with and without AD. The analysis included 335 stool samples collected from 102 unique participants, with some individuals providing multiple samples over a period of five months or more. This dataset includes older adults with Alzheimer’s disease and healthy controls, but does not include other types of dementia or individuals with mild cognitive impairment.

### A. CLINICAL DATA

**FIGURE 1.**
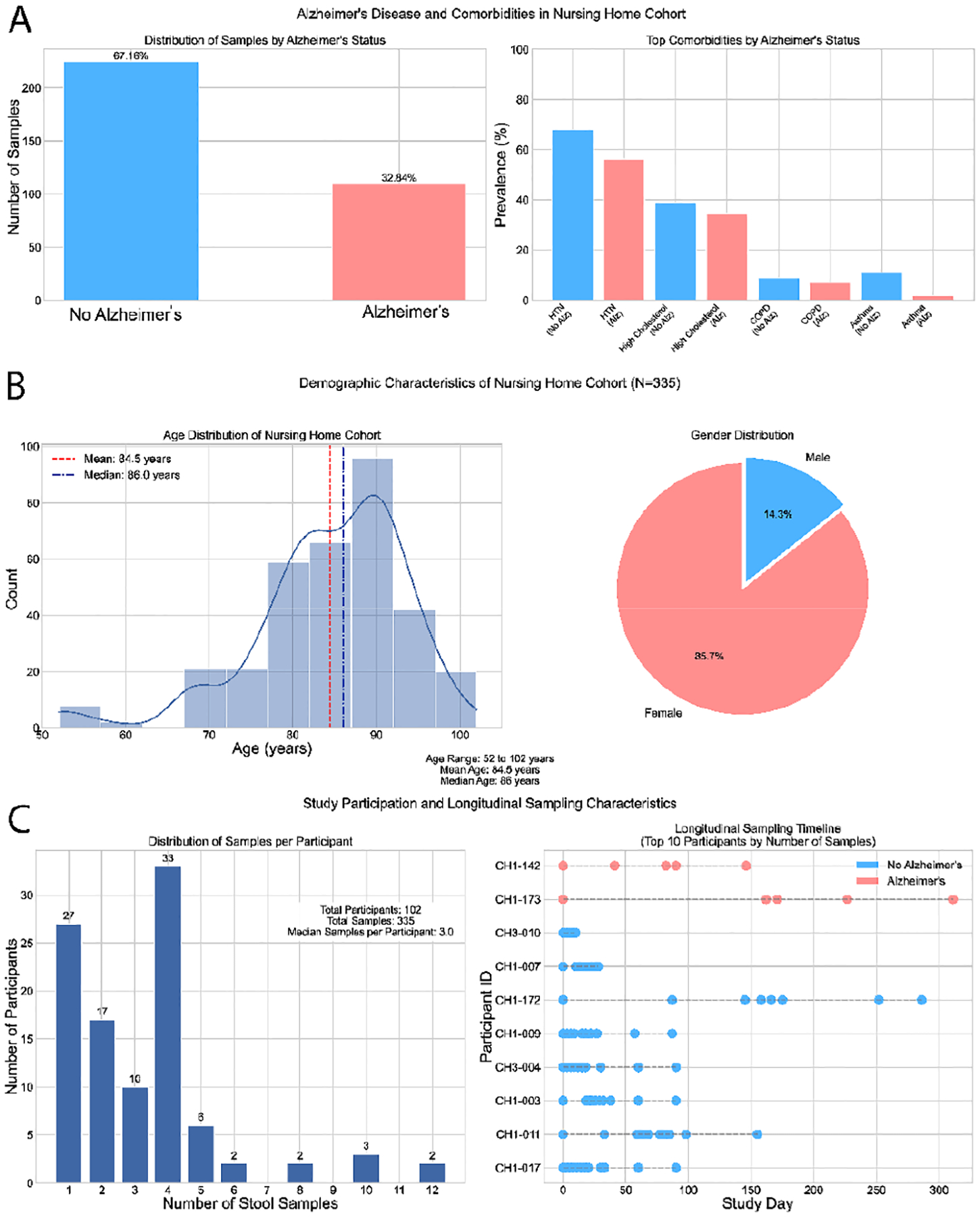
The Nursing Home Cohort Clinical Data Description (source: [28]). (A) Bar plots illustrate the distribution of participants based on Alzheimer’s status and the prevalence of key comorbidities. 67.16% of participants do not have Alzheimer’s disease, while 32.84% do. The most prevalent comorbidities include hypertension (HTN), and various cardiovascular diseases (CVD), with prevalence categorized by Alzheimer’s status. (B) The demographic characteristics of the cohort (N=335) encompass age distribution, with a mean age of 84.5 years and a median age of 86.0 years, alongside a pie chart depicting gender distribution, which shows a predominance of females (85.7%) in the cohort. (C) Study participation metrics include the distribution of stool samples per participant and a longitudinal sampling timeline for the top 10 participants based on the number of samples collected. The median number of samples per participant is 3. Alzheimer’s status is color-coded across all visualizations.

Within the clinical dataset, each sample was annotated with detailed clinical metadata, including AD status, comorbidities, demographic information, and longitudinal sampling data. Of the total sample, 32.84% came from individuals diagnosed with AD, while the remaining 67.16% came from individuals without dementia. The cohort is predominantly female (85.7%), with a mean age of 84.5 years and a median of 86.0 years, spanning an age range from 52 to 102 years. This age distribution reflects the demographic characteristics typical of long-term care populations (FIGURE 1).

In addition to cross-sectional clinical features, the dataset included a longitudinal sampling component. Participants provided between one and 12 stool samples, with a median of three samples per participant, facilitating within-subject comparisons over time. Sampling timelines varied by individual, with consistent representation across the AD and non-AD groups. This temporal resolution supports dynamic analyses of microbiome profiles and disease status. The integrated design of the dataset, linking clinical diagnoses, demographic characteristics, comorbid conditions, and longitudinal biospecimens, provides a robust framework for exploring the complex interactions between neurodegenerative diseases, aging, and host-associated microbial communities. Ensuring data quality and biological relevance, our biologists addressed missing values and performed initial feature selection based on domain-specific biological considerations during the preprocessing stage. These clinical features were integrated into the reasoning and analytical processes of the ADAM framework.

### B. GUT MICROBIOME DATA

The original microbiome dataset was generated through a standardized process, beginning with collecting stool samples from nursing home residents, followed by DNA extraction using a Qiagen DNeasy PowerSoil Pro Kit. The resulting DNA was used to create a pool containing 2 nM DNA, 12μL RSB with Tween, and 4μL of diluted PhiX prep, which was then pipetted into a P4 Illumina flow cell cartridge. The sequencing run was generated on the BaseSpace Illumina platform and validated using Illumina NextSeq 2000 prior to analysis. Post-sequencing, the dataset underwent prevalence and abundance-based filtering to reduce technical noise and enhance biological interpretability. Bacterial species were retained if they appeared in more than 5% of samples (i.e., prevalence > 17 samples) and exhibited a relative abundance greater than 0.01% of the total community (i.e., proportion > 1e-4). This procedure reduced the dataset from 940 to 247 species, focusing subsequent analyses on taxa consistently represented across the cohort.

**FIGURE 2.**
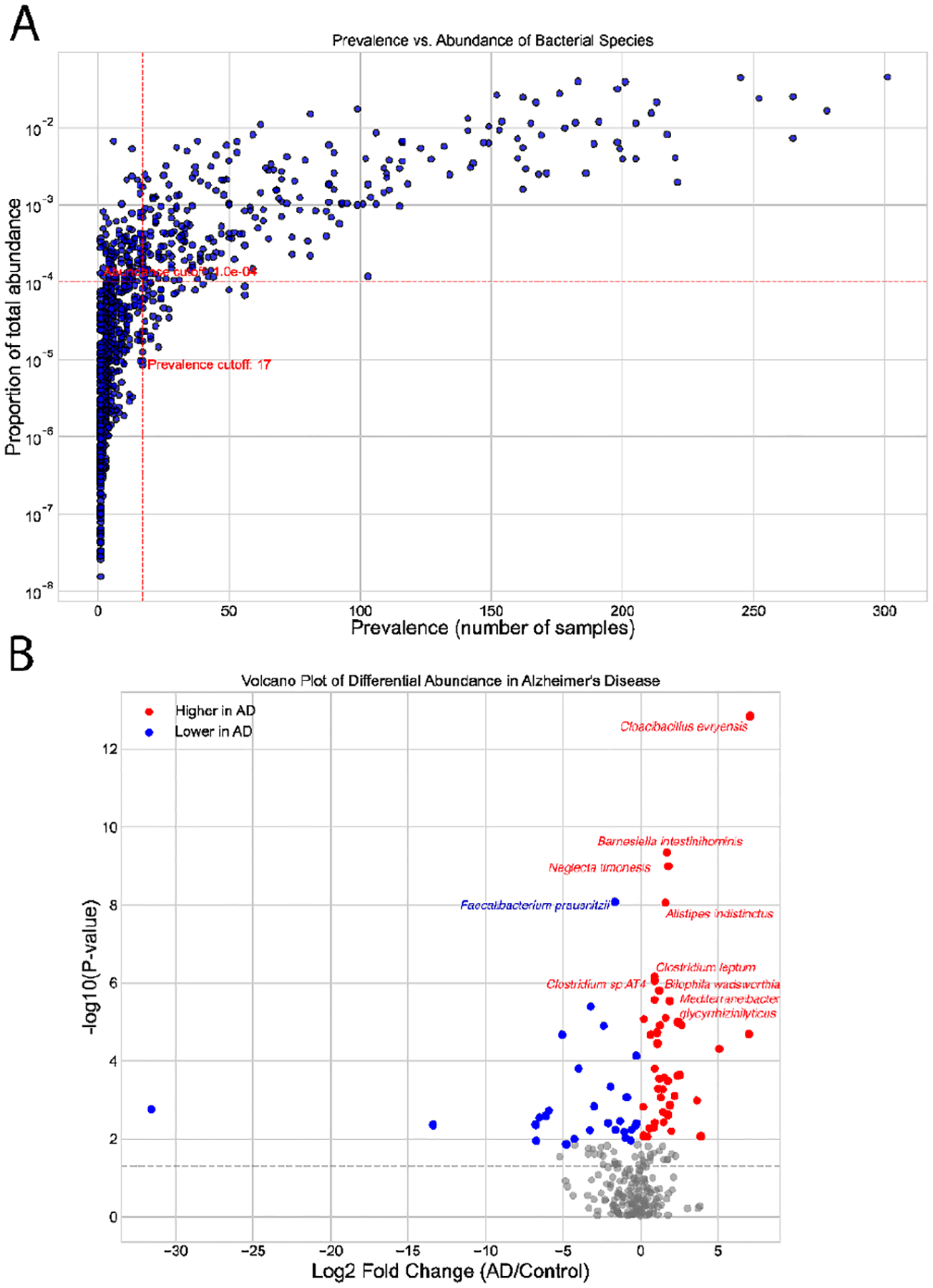
Nursing Home Cohort Bacteria Data Description (source: [28]). (A) Prevalence vs. Abundance of Bacterial Species: Scatter plot illustrating bacterial species by their prevalence (the number of samples in which each species appears) and proportional abundance across the dataset. Red dashed lines represent the filtering criteria applied during preprocessing: species present in fewer than 5% of samples (prevalence cutoff: 17) or with a total relative abundance below 1e-4 were removed to minimize noise and focus on biologically relevant taxa. (B) Volcano Plot of Differential Abundance: log_2_ fold change versus –log_10_ (p-value) for all bacterial species. Species with significantly higher abundance in Alzheimer’s patients are shown in red; those lower in AD are shown in blue.

We characterized the dataset by analyzing species-level prevalence, abundance, and differential abundance metrics, as shown in FIGURE 2. The prevalence-abundance distribution (FIGURE 2(A)) exhibits a typical long-tailed pattern, where most bacterial species show low prevalence and low proportional abundance. These rare taxa often add to noise and variance, reducing statistical power. To address this issue, we implemented filtering thresholds, retaining only species present in at least 5% of samples (prevalence ≥17) and with a total abundance greater than 1e-4. This approach ensures that our analysis focuses on reproducible and biologically relevant species.

After preprocessing, we analyzed differential abundance to pinpoint taxa significantly linked to AD status. The volcano plot in FIGURE 2(B) displays log_2_ fold changes versus –log_10_ (p-values), indicating species that are higher in AD (red) and those that are lower (blue). This thorough profiling approach improves the signal-to-noise ratio, ensuring that only reliable microbial features contribute to the ADAM framework. Other data types, like neuroimaging and peripheral biomarkers, were not included in this study.

## III. ARCHITECTURE OF ADAM

**FIGURE 3.**
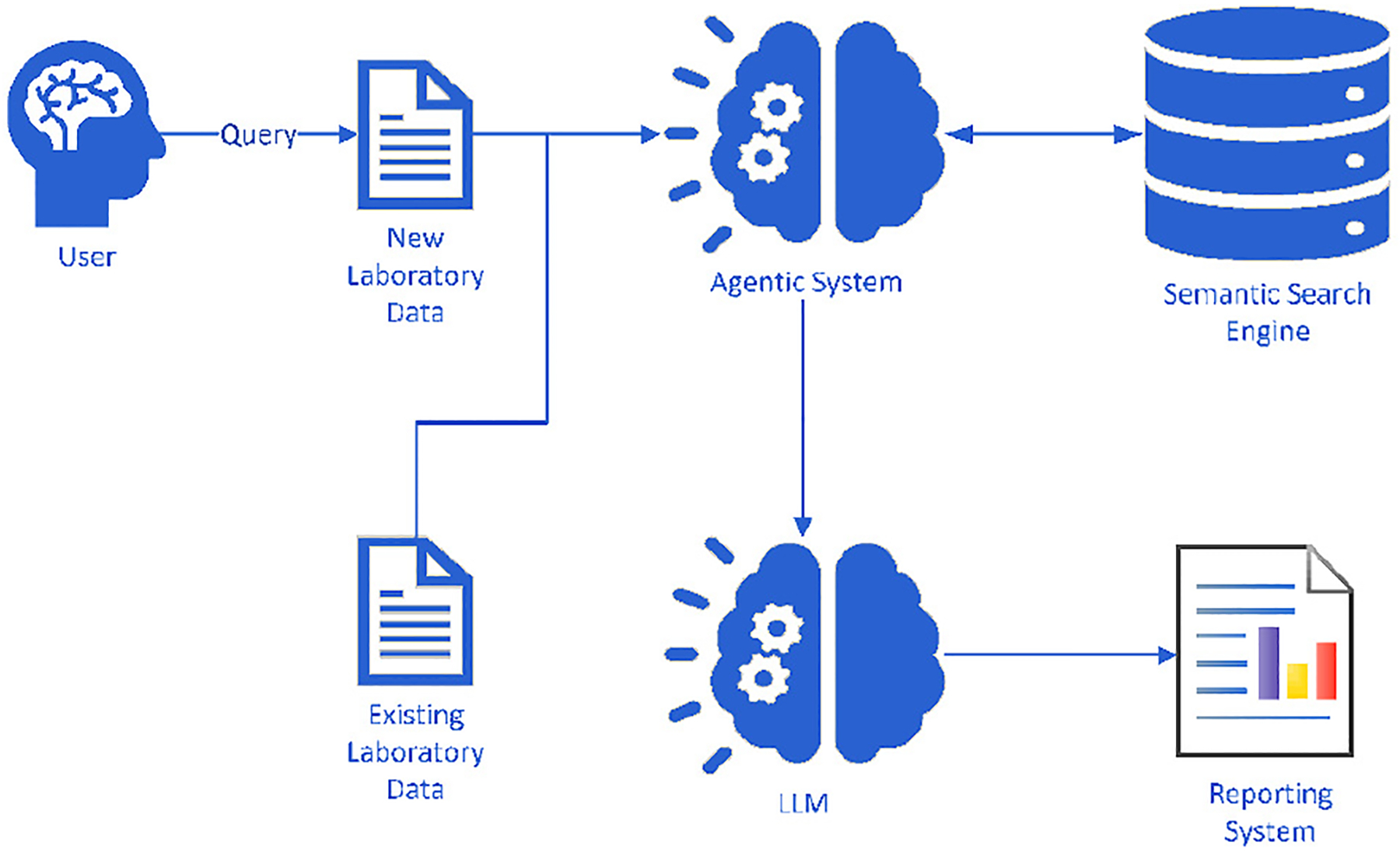
Workflow of the ADAM Framework. This diagram illustrates user interactions within the ADAM system. When a user submits a query along with new laboratory data, the framework employs the agentic system to refine the query and identify relevant literature evidence from a semantic search engine, integrating it with existing laboratory data. The semantic search engine collaborates with the agentic system, alongside the LLM, to reason, analyze, and interpret data, ultimately delivering precise analytical reports.

The ADAM framework comprises an agentic system, a semantic search engine, two base LLMs, and a reporting module, along with new and existing laboratory data. FIGURE 3 illustrates the architecture of the ADAM framework. The ADAM framework begins with users inputting new data into the agentic system. The agentic system processes the new data in its computational agent and sends results to the summarization and classification agents, along with historical laboratory data. This information is then processed through a semantic research engine and the base LLMs, with cosine similarity controlling the return quality. Finally, a reporting system presents the output as a classification report.

### A. AGENTIC SYSTEM

**FIGURE 4.**
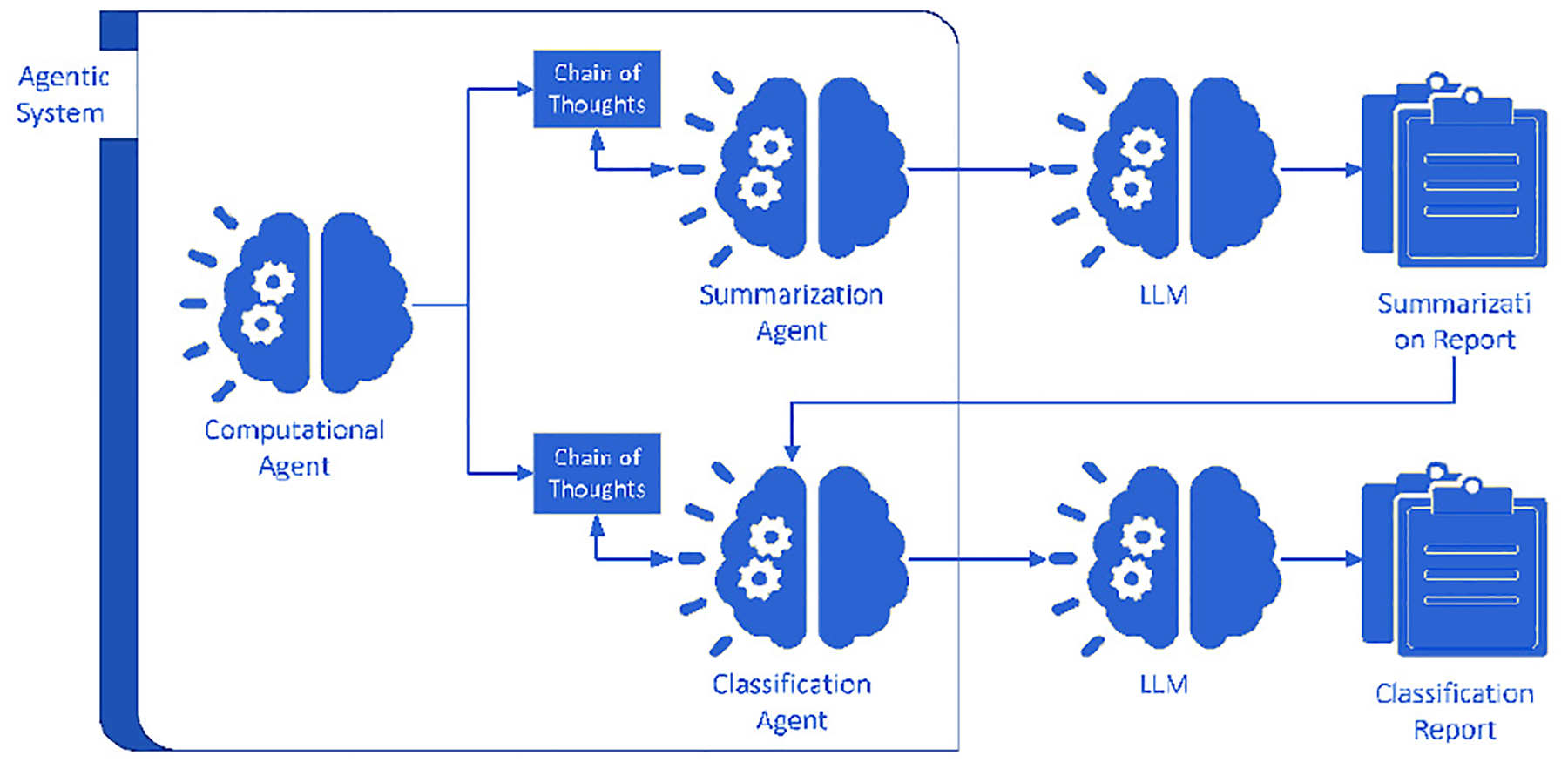
Agentic System Workflow. The Diagram illustrates the coordinated interaction among three AI agents–Computational, Summarization, and Classification–within the agentic system. Each agent leverages Chain-of-Thought reasoning and communicates with the LLM to generate structured outputs. The system collectively produces Alzheimer’s-specific summarization and classification reports by integrating computational analysis with contextual interpretation.

The agentic system within the ADAM framework is an AI architecture composed of three distinct yet interconnected agents that work collaboratively to perform specialized tasks toward a common analytical goal: a computational agent, a summarization agent, and a classification agent. This multi-agent design produces both computational outputs and semantic outputs that are informed by computational analyses and peer-reviewed literature, thereby enhancing analytical strength while reducing hallucinations and the likelihood of misclassification inherent in the base LLMs. These agents transform clinical and metagenomic data into interpretable patient-specific insights. As illustrated in FIGURE 4, the computational agent processes the newly uploaded data to extract key features and analytical outputs. These outputs, along with historical laboratory data, are then used for summarization with a proposed context window size of approximately 100,000 tokens and classification with a proposed context window size of approximately 50,000 tokens, guided by CoT reasoning and supported by LLMs. The summarization agent generated context-aware narratives, whereas the classification agent determined the AD status.

#### 1) BASE LLM SELECTION RATIONALE

GPT-4o and its light variant GPT-4o-mini were selected as the base LLM for ADAM-1 due to their consistently superior performance across a wide range of biomedical and clinical benchmarks. In cancer genomics, GPT-4o achieved the highest accuracy (0.7318) when classifying clinically actionable variants from multiple knowledge bases, outperforming Qwen 2.5 and Llama 3.1, and showing increased concordance with expert annotations even in complex evidentiary contexts [29]. In medical education, GPT-4o demonstrated top-tier performance, achieving an accuracy of 89.2% on the Japanese National Medical Examination (JNME), significantly exceeding Claude 3 Opus, Gemini 1.5, and earlier GPT versions [30]. It was notably reliable for easy-to-moderate tasks, reflecting its capacity for high factual retention and instructional consistency. Additionally, GPT-4o achieved the highest accuracy across multiple medical licensing examinations, including the United States Medical Licensing Examination (USMLE), the Professional and Linguistic Assessments Board (PLAB), the Hong Kong Medical Licensing Examination (HKMLE), and the National Medical Licensing Examination (NMLE), demonstrating its robustness across diverse cultural and linguistic contexts in medicine [31]. These empirical advantages directly support its integration into ADAM-1, where high-stakes reasoning on clinical and microbiome data requires both precision and stability for the summarization and classification agents in biological studies.

#### 2) COMPUTATIONAL AGENT

The computational agent comprises bioinformatics and machine learning. Alpha diversity (Shannon, Simpson, Berger-Parker dominance) and beta diversity (Bray-Curtis, Jaccard, Canberra) were calculated to provide a descriptive analysis of bacterial relationships. XGBoost [32] was chosen as the primary machine learning model, working in conjunction with SHAP [33] to generate feature importance information and SHAP values that describe feature interactions in relation to AD status from a global and an individual study subject perspective. The formula for the computational agent is as follows:

(1) $${\text{CA}}_{\text{comp}}(\text{MD},\text{CF})\;=\text{SHAP}(\text{XGB}(\text{MD},\text{CF})),{\text{D}}_{\alpha }(\text{MD}),{\text{D}}_{\beta }(\text{MD})$$

where:

${\text{CA}}_{\text{comp}}$ is the Computational Agent responsible for processing microbiome and clinical data to generate interpretable outputs.

MD is the Microbial Data input (e.g., microbial abundance profiles).

CF is a clinical feature input (e.g., patient demographics and health records).

SHAP(XGB(MD, CF)) represents the XGBoost model embedded within SHAP, which enables explainable machine learning via feature attributes and interactions.

${\text{D}}_{a}(\text{MD})$ denotes the Alpha diversity metrics (Shannon, Simpson, Berger-Parker dominance) computed from microbial data.

${\text{D}}_{\beta }$ (MD) denotes the Beta diversity metrics (Bray-Curtis, Jaccard, Canberra) representing microbial community dissimilarity.

#### 3) SUMMARIZATION AGENT

The summarization agent integrates the computational agent’s results, CoT reasoning, semantic search engine, and GPT-4o model to provide an overall context summary. The model demonstrates superior language understanding and coherence in long text [34]. Its ability to maintain contextual relevance and accuracy in summaries is well documented, particularly in academic and technical domains. This makes it an ideal choice for summarization tasks that require precision and reliability. The formula for the summarization agent is as follows:

(2) $${\text{SA}}_{\text{summary}}\left({\text{CA}}_{\text{comp}},{\text{CoT}}_{\text{reasoning}},{\text{SS}}_{\text{search}},{\text{LLM}}_{\text{GPT}-4\text{o}}\right)\;={\text{LLM}}_{\text{GPT}-4\text{o}}\left(\text{Integrate}\left({\text{CA}}_{\text{comp}},{\text{CoT}}_{\text{reasoning}},{\text{SS}}_{\text{search}}\right)\right)$$

where:

- ${\text{SA}}_{\text{summary}}$ (*Summarization Agent*): Synthesizes context-aware insights.
- ${\text{CA}}_{\text{comp}}$ (*Computational Agent*): Processes microbial and clinical data to generate structured outputs.
- ${\text{CoT}}_{\text{reasoning}}$ (*Chain-of-Thought reasoning*): Provides structured logical interpretive paths.
- ${\text{SS}}_{\text{search}}$ (*Semantic Search*): Retrieves relevant contextual information using RAG from a vector database.
- ${\text{LLM}}_{\text{GPT-4o}}$ (*Large Language Model*): GPT-4o model that generates accurate, coherent summaries.
- Integrate(⋅): Combines multiple sources into a unified semantic context.

We propose a logic for CoT reasoning for the summarization agent in the following eight steps to ensure a comprehensive understanding of the input:

1. Patient Overview: Starts with basic patient demographics and general health status
2. Key Clinical Markers: Identifies important biomarkers or health indicators
3. Gut Microbiome Profile: Analyzes the specific microorganisms present in the gut
4. Diversity Metrics Analysis: Evaluates biodiversity measures of the gut microbiome
5. Interactions and Mechanisms: Examines relationships between the microbiome and clinical markers
6. Descriptive Correlation: Identifies statistical relationships without implying causation
7. Machine Learning analysis and probabilistic assessment: ML models were used to evaluate the patterns and make probabilistic predictions
8. Final Comprehensive Descriptive Summary: Creates a holistic summary integrating all findings

#### 4) CLASSIFICATION AGENT

The classification agent integrates the output of the computational agent, the output of the summarization agent, CoT reasoning, our semantic search engine, and the GPT-4o-mini model for content classification. We used GPT-4o-mini for AD classification tasks because it efficiently processes extremely long texts, delivers high accuracy and reliability essential for medical diagnosis, and offers unmatched cost-effectiveness compared with similar models [35]. The formula for a classification agent is as follows:

(3) $${\text{CA}}_{\text{class}}\left({\text{CA}}_{\text{comp}},{\text{SA}}_{\text{summary}},\right.\;\left.{\text{CoT}}_{\text{reasoning}},{\text{SS}}_{\text{search}},{\text{LLM}}_{\text{GPT}-4\text{o}-\text{mini}}\right)\;={\text{LLM}}_{\text{GPT}-4\text{o}-\text{mini}}\left(\text{Integrate}\left({\text{CA}}_{\text{comp}},\text{SA},{\text{CoT}}_{\text{reasoning}},\right.{\text{SS}}_{\text{search}}))\right.$$

Where:

- ${\text{CA}}_{\text{class}}$ (*Classification Agent*): Responsible for generating Alzheimer’s disease classification outputs.
- ${\text{CA}}_{\text{comp}}$ (*Computational Agent*) contains structured data features (e.g., predictions and feature importance).
- ${\text{SA}}_{\text{summary}}$ (*Summarization Agent*): Provides high-level contextual insight.
- ${\text{CoT}}_{\text{reasoning}}$ (*Chain-of-Thought reasoning*): Guides interpretability through logical step-by-step inference.
- ${\text{SS}}_{\text{search}}$ (*Semantic Search*): retrieves conceptually relevant knowledge from a vector-based index.
- ${\text{LLM}}_{\text{GPT}-40-\text{mini}}$ (*Large Language Model*): Optimizes for fast and efficient classification tasks.
- Integrate (⋅) is a function that merges multiple contextual and data-driven sources into a unified representation.

We propose the CoT reasoning logic for the classification agent in the following eight steps to ensure robustness in the classification task.

1. Historical Data Insights: Analyzes past patient data to establish baselines and trends
2. Diversity Metrics & Classification Refinement: Uses microbiome diversity measures to improve classification accuracy
3. Adaptive Threshold Decisioning: Determines dynamic thresholds for classification rather than fixed values
4. Handling Edge Cases & Misclassifications: Identifies and addresses outliers or difficult-to-classify cases
5. Comprehensive Summary of this Visit: Provides a complete analysis of the current patient data
6. SHAP Feature Importance: Uses SHAP values and ML outputs to understand the features that influence the model’s predictions most
7. Key Considerations for Prediction and Misclassification Adjustments: Identifying factors that might lead to misclassification and how to adjust for them
8. Prediction Decision Rules: Establishes clear rules for making final classification decisions

### B. BASE LLMS

This study utilized two OpenAI LLMs: GPT-4o (2024-11-20) for summarization and GPT-4o-mini (2024-07-18) for improved speed and cost efficiency. Their selection aligns with the design concepts of the summarization and classification agents. Using an eight-step CoT reasoning logic, the summarization agent is designed to handle more contextual data, including single-visit and longitudinal data. Each step corresponds to a relevant RAG output, explaining the meaning of each step in summarizing an individual’s clinical conditions and microbiome profiles. The summarization task was designed to process approximately 100,000 tokens of text data at a time, given its superior ability to handle clinical text summarization [36]. Therefore, as a downstream counterpart to GPT-4o, GPT-4o-mini was proposed for focused binary classification tasks utilizing a dedicated CoT reasoning framework for classification. It processes data that has been preprocessed by computational and summarization agents powered by GPT-4o and is optimized for high-throughput classification over approximately 50,000 tokens per instance.

### C. SEMANTIC SEARCH ENGINE

The knowledge base comprised 76,751 full-text publications or abstracts programmatically retrieved in October 2024 from PubMed Central (PMC) using the NCBI Entrez system via E-utilities and Entrez Direct. Articles were identified based on user-defined query terms applied to specific fields, such as titles, abstracts, or text words, and retrieved in XML format using the efetch utility. The collected records were subsequently processed using an embedding model and indexed into two vector databases to support downstream retrieval tasks.

**FIGURE 5.**
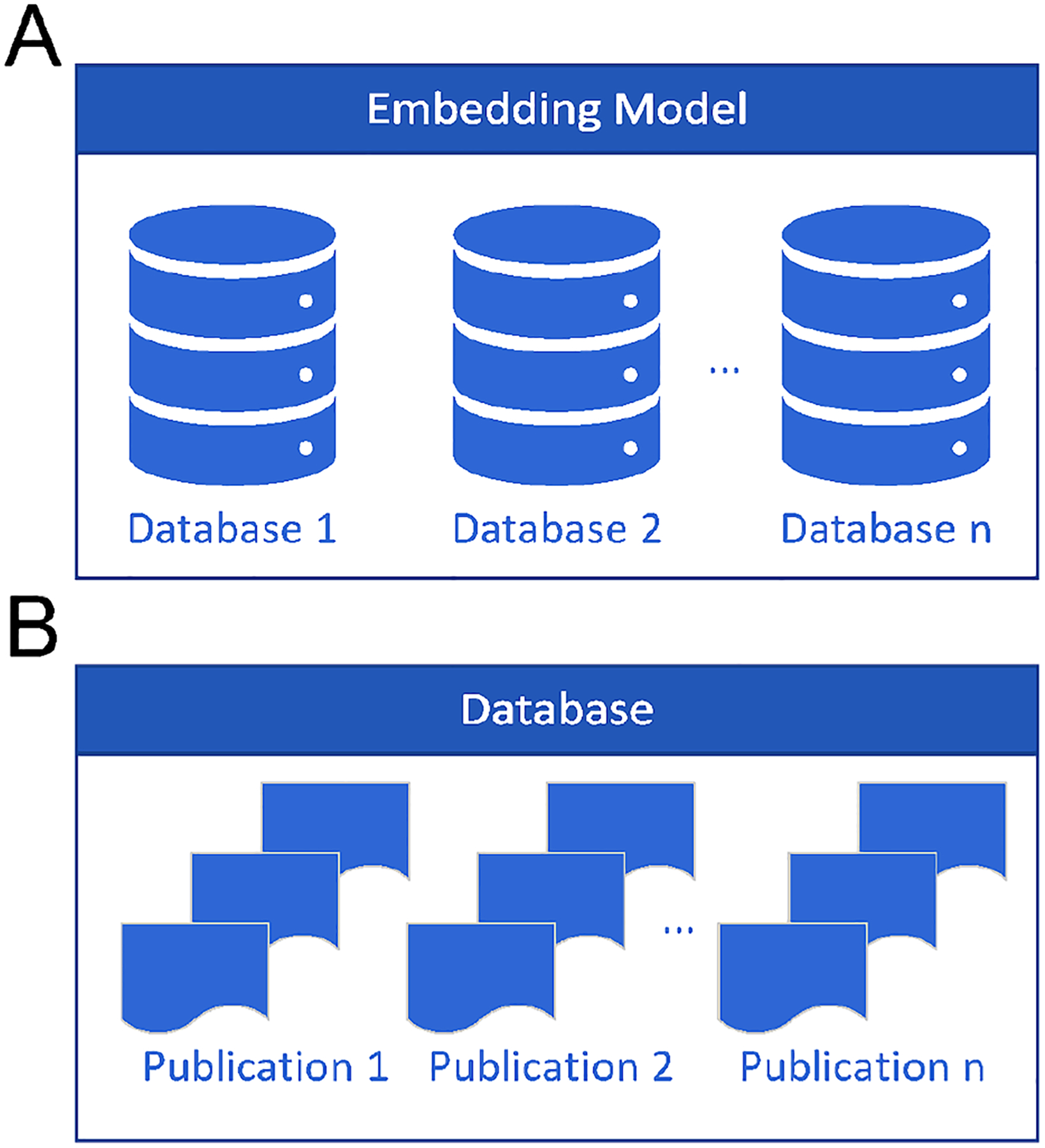
Semantic Search Engine Architecture. Semantic Search Engine. (A) The embedding model converts documents into and out of numerical vectors. (B) Vector databases work in parallel with the semantic search engine that returns the most relevant information measured by cosine similarity. Inside a Vector Database. Each publication is split into 2000-character vector chunks, with a 20 percent overlap between the prior chunk of text and the next.

The publications were divided into 2,058,502 text chunks, each containing 2,000 characters, with a 20 percent overlap with the previous chunk to ensure continuity and preserve context. This approach minimizes the risk of losing critical information when analyzing the AD-related literature. The embedding model converts these chunks into high-dimensional vector representations, which are stored in two separate vector databases optimized for semantic search and retrieval (FIGURE 5). These databases enable rapid querying of relevant AD research, supporting tasks such as disease classification, summarization, and hypothesis generation within the ADAM framework.

#### 1) PUBLICATIONS

This component serves as a source of existing literature and the reasoning logic of the ADAM, integrating 76,751 publications relevant to AD research at the time of this study. The keywords used for indexing included Alzheimer’s disease, Gut-Brain Axis, Gut Microbiome, and Immunosenescence (TABLE 1).

Each publication $P_{i}$ is indexed by a set of relevant keywords $K_{i,j}$, forming a keyword-embedding vector $K\left(P_{i}\right)$:

(4) $$K\left(P_{i}\right)=\sum_{j=1}^{m}w_{i,j}\cdot \text{EmbeddingModel}\left(K_{i,j}\right)$$

where:

$P_{i}$ represents the i-th publication.

$K_{i,j}$ is the j-th keyword associated with $P_{i}$.

$w_{i,j}$ is the weight assigned to $K_{i,j}$ based on its relevance to $P_{i}$.

$K\left(P_{i}\right)$ is the aggregated embedding vector representing the keywords of $P_{i}$, facilitates its retrieval from the knowledge base.

The vector $K\left(P_{i}\right)$ enables the efficient retrieval of publications based on semantic relevance to query keywords in AD research.

#### 2) EMBEDDING MODEL

The embedding model transforms textual data into embeddings, thereby allowing the framework to process and effectively retrieve relevant information. We chose text-embedding-ada-002 because of its high-cost effectiveness and proven success in text processing since its release in December 2022. To determine the most suitable embedding models for this study, we compared three available embedding models from OpenAI: text-embedding-ada-002 (hereafter referred to as ada-002), text-embedding-3-small (hereafter referred to as 3-small), and text-embedding-3-large (hereafter referred to as 3-large) to determine the most suitable embedding models for this study [37]. These three models were evaluated based on the following criteria: semantic richness, computational efficiency, storage requirements, cost-effectiveness, versatility, and adoption (TABLE 2).

Furthermore, we applied these three embedding models to a small-scale test vector database derived from gut microbiome publications, where each text chunk contained 2,000 characters and had a 20 percent overlap with the previous chunk, utilizing a cosine similarity threshold of 0.7. Our empirical findings indicated that the ada-002 model yielded more consistent results within the LLM and vector database configurations during the RAG retrieval process.

Given an input text T, the embedding model generates an embedding vector $\vec{E}$ as follows:

(5) $$\vec{E}=\text{EmbeddingModel}\;(T)$$

where:

T represents the i-th publication.

$\vec{E}$ is the resulting embedding vector in high-dimensional space.

This embedding vector $\vec{E}$ captures semantic and contextual information from T, facilitating effective retrieval and matching within the framework.

#### 3) VECTOR DATABASES

When embedding publications into a vector database, the publication text is divided into 2000-character segments, with a 20 percent overlap between segments, to maintain the information flow and provide a more continuous and coherent representation of the text.

Given:

Segment length s=2000 characters

Overlap o=0.2×s=400 characters

Effective step size=s-o=1600 characters

(6) $$p_{i}=1+(i-1)\times (s-o)$$

This formula calculates the starting position of the i-th segment:

Segment 1 starts at position 1

Segment 2 starts at position 1601

Segment 3 starts at position 3201, and so on…

Total number of segments n required to cover the entire text L, each new segment after the first adds (s-o)=1600 new characters. Subtraction of o from the numerator accounts for its overlap with the last segment.

(7) $$n=\left\lceil \frac{L-o}{s-o}\right\rceil$$

For example:

If L=5800 characters

First segment covers positions 1–2000

Second segment covers positions 1601–3600

Third segment covers positions 3201–5200

Fourth segment covers the position of 5211–7200

Using the original formula $n=\lceil \frac{5800-400}{2000-400}\rceil =\lceil \frac{5400}{1600}\rceil =\lceil 3.375\rceil =4$, as a result, 5800 characters need 4 segments.

This configuration incorporated a 20 percent overlap between consecutive segments to maintain continuity in the embeddings. Consequently, the vector databases in the semantic search engine comprised 2,058,502 discrete overlapping segments derived from 76,751 publications (TABLE 1).

## IV. HARDWARE, SOFTWARE, AND COMPUTATIONAL COST

The experiments were conducted on an Ubuntu 24.04.2 LTS workstation equipped with an Intel^®^ Core^™^ i9–10900X (20 threads), 128 GB RAM, and four NVIDIA GeForce RTX^™^ 3090 GPUs, providing a robust computing environment for LLM and machine learning tasks. The software stack was built on Python 3.10.14, utilizing XGBoost 2.1.3, and Scikit-Learn 1.5.2 for model training, with Optuna 4.1.0 handling hyperparameter optimization.

For LLM processing, the system integrates OpenAI 1.55.1, PandasAI 2.4.2, LangChain 0.3.8, and LangChain-Chroma 0.1.4. Additionally, Scikit-Bio 0.6.2, SciPy 1.10.1, NumPy 1.26.4, and Pandas 1.5.3 facilitated data processing and analysis. For visualization and interpretability, the setup included Matplotlib 3.7.5, Seaborn 0.12.2, and SHAP 0.46.0. This configuration ensures high computational efficiency and scalability for deep learning workflows.

Within the ADAM framework, the summarization agent typically requires 2 to 5 minutes per observation, while the classification agent takes approximately 5 to 10 minutes, depending on the availability of the OpenAI API. XGBoost, optimized using Optuna, consumes approximately 310 to 350 MB of VRAM on an NVIDIA 3090 GPU and completes training and testing in 2 to 3 minutes. For cost transparency, we also report token pricing: GPT-4o currently costs $2.50 per million input tokens and $10.00 per million output tokens, while GPT-4o-mini costs $0.15 per million input tokens and $0.60 per million output tokens [38].

## V. DATA SPLIT AND SEEDING POLICY

### A. DATA SPLIT POLICY

We implemented two data-splitting policies: one for selecting the baseline model and the other for the ADAM framework. In the baseline model selection phase, the data were initially split at 75:25 by a unique Study ID and stratified according to the AD status. In the second phase, which involved the ADAM framework, 75% of the data was retained as the training or reference dataset. For testing, we randomly selected 15 positive and 15 negative cases from the 25% portion owing to hardware limitations. This approach allowed us to work efficiently with the LLM while maintaining statistical significance (n=30).

### B. SEEDING AND MEASURES

We implemented two seeding strategies to support the different phases of the study: one for baseline model selection and the other for creating the ADAM framework. The initial phase involved applying 10 random seeds to identify the best-performing classifier based on the accuracy, AUC, and F1 score. The selected model was then integrated into ADAM, a language-model-based system designed to enhance the binary classification of AD. A broader evaluation was conducted using 30 random seeds to examine whether ADAM improved the mean F1 score and reduced the performance variance compared with the baseline. This seeding policy design ensured that the performance metrics in both phases were derived from stable and reproducible evaluations, allowing for a fair assessment of ADAM’s added value over the baseline model.

## VI. BASELINE MODEL SELECTION

We trained three classifiers on the AD dataset: XGBoost, random forest, and logistic regression. These models are commonly used in biological studies and are computationally efficient for laboratory computers. The best-performing model was selected as the baseline for this study. The model selection process involved two phases: feature selection and model training, both optimized using Optuna, which is a Bayesian-based hyperparameter-tuning framework [39]. To identify the most relevant features, we configured an XGBoost-based feature selector applied to all three machine learning models. The selected features are subsequently fed into the proposed models for further training and testing.

**FIGURE 6.**
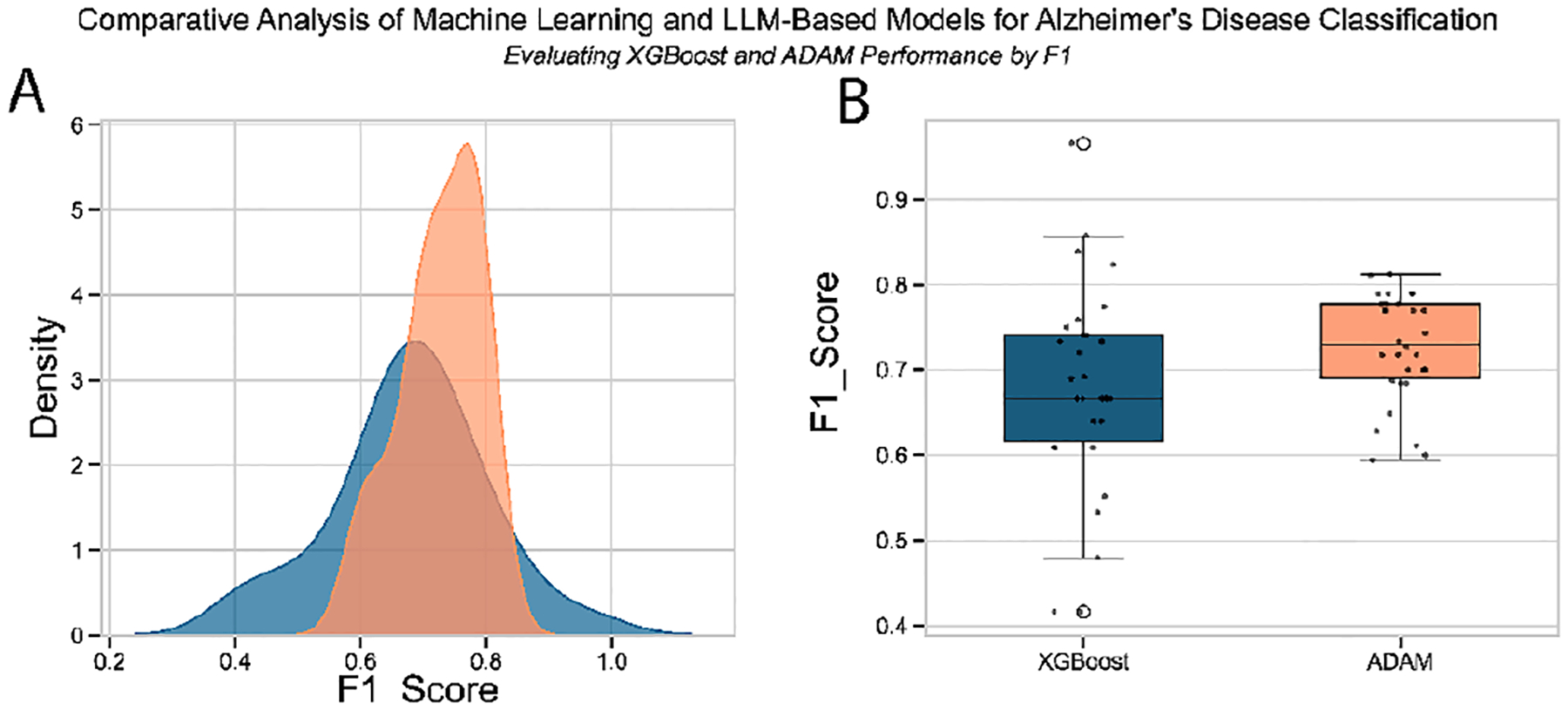
Comparative Analysis of XGBoost and ADAM. (A) Density plots and (B) boxplots of F1 scores comparing XGBoost and the ADAM framework across multiple runs. ADAM demonstrates a higher median F1 score with reduced variance, suggesting more consistent and reliable classification performance for AD prediction.

We implemented several performance metrics to evaluate the model’s effectiveness, including accuracy, AUC, F1 score, and overall performance index. Each metric was calculated by averaging the results from 10 different random seeds, which helped capture the variability and enhance the robustness of the evaluation process. The results are shown in FIGURE 6 and TABLE 3, which summarizes the comparative performance of each model across all metrics. By relying on multiple evaluation criteria, we aimed to assess not only how well each model classifies, but also how reliably and consistently it performs across different data splits, which is especially critical in clinical datasets where precision and balance are essential.

As shown in TABLE 3, XGBoost consistently outperformed logistic regression and Random Forest across all key metrics. It achieved the highest average accuracy, AUC, and F1 score, demonstrating its superior discriminative ability and robustness. Furthermore, XGBoost exhibited the highest stability across different seeds, underscoring its reliability in managing complex and heterogeneous data related to AD. Owing to its strong performance and consistency, XGBoost was selected as the baseline model in this study. This served as the foundation against which the proposed ADAM framework was assessed, providing a high-performance and dependable benchmark for future model comparisons.

## VII. RESULTS AND EVALUATION

### A. EVALUATION STRATEGY

The evaluation strategy aimed to determine whether ADAM outperformed the baseline XGBoost model in binary classification tasks using the F1 score. The F1 score is directly affected by false negatives (FN) because it is the harmonic mean of precision and recall, with recall particularly sensitive to false negatives [40]. This is especially critical in medical applications, where false negatives can lead to severe consequences [41], [42]. In the context of AD, false negatives can result in delayed diagnosis, leading to inadequate monitoring and treatment, loss of social and financial benefits, increased emotional distress for patients and caregivers, and ultimately, worse patient outcomes [43], [44], [45]. The mean F1 score quantifies the overall predictive performance, whereas the variance of the F1 score assesses the consistency and stability of the ADAM performance compared to XGBoost.

Each model was fine-tuned using Optuna, with extensive cross-validation. For each of the 30 seeds, we conducted 200 Optuna trials using Bayesian optimization (TPE sampling), with each trial evaluated via stratified 3-fold cross-validation, resulting in 600 evaluations per seed. The best hyperparameters identified for each seed were used exclusively for that seed’s final model, and the corresponding F1 scores were recorded. The statistical significance of the difference in mean F1 scores between ADAM and XGBoost was analyzed using the Mann-Whitney U test, a non-parametric test that does not require normality assumptions [46]. Additionally, an F-test was conducted to compare the variances in the F1 scores, providing insights into the performance stability of each model [47].

### B. F1 SCORE AND VARIABILITY ANALYSIS

Following the evaluation strategy, we conducted a comparative analysis of ADAM and XGBoost based on 30 independent experimental runs. The focus was on two core aspects of model performance: accuracy, measured by the mean F1 score, and stability, indicated by the variability of F1 scores across runs. By assessing central tendency and dispersion, we determined which model performed better on average and which produced more consistent results.

Across these runs, ADAM achieved a higher mean F1 score (0.7263) than XGBoost (0.6774), as determined by the Mann-Whitney U test (p = 0.0418), indicating a statistically significant improvement at the 95% confidence level. In addition, ADAM demonstrated a lower standard deviation (0.0632) than XGBoost (0.1217), as supported by Levene’s test (p = 0.0300), indicating a more stable and consistent performance. The medium effect size (Cohen’s d = 0.5038) further underscores the practical relevance of ADAM’s superior and reliable classification capability.

**FIGURE 7.**
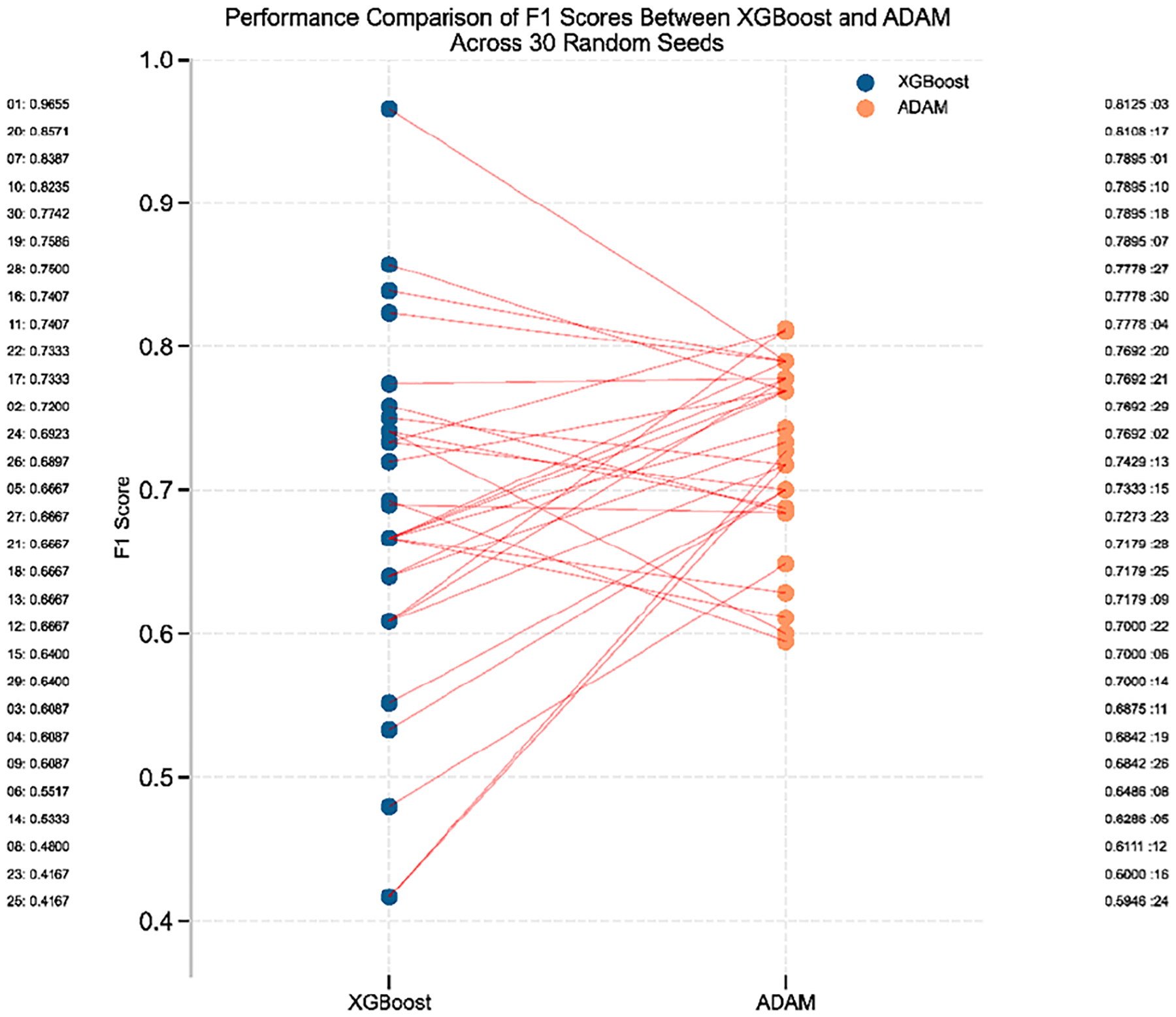
Performance Comparison of F1 Scores Between XGBoost and ADAM. Line plot comparing F1 scores of XGBoost and ADAM across 30 random seeds. Each line connects paired runs for a given seed. ADAM consistently shows improved or more stable F1 performance relative to XGBoost, highlighting its robustness and reduced variability across repeated evaluations.

The box plots and density distributions (FIGURE 6) demonstrate that ADAM maintains a narrower F1 score distribution (FIGURE 6(B)) with a higher median and fewer extreme values than XGBoost. Although both models exhibit similar central performance around 0.7, the density plot (FIGURE 6(A)) reveals that ADAM’s F1 scores cluster more tightly around 0.75, whereas XGBoost displays a broader spread, ranging from 0.4 to nearly 1.0, indicating greater performance variability across different runs. This higher consistency in the ADAM performance suggests that it may offer more reliable predictions for AD classification (FIGURE 7).

These findings collectively demonstrate that ADAM significantly outperforms XGBoost, offering an improved average performance and greater consistency and stability across experimental runs.

## VIII. REPORTING MODULE

ADAM is not only a classifier but also a reporting system. Its classification is based on the combined results of the three AI agents presented in textual form. Its logical reasoning design classifies the AD status while generating analytical reports for each study subject. We listed two sample classification reports as follows:

### A. SAMPLE REPORTS

TABLE 4 presents two sample reports produced by ADAM, one for a positive case and one for a negative case. Due to the length of the full report, only the conclusion section is shown for each. The complete set of over 900 ADAM-generated case-specific classifications is available at https://github.com/melhzy/ADAM/tree/main/reporting.

Analyses of samples FB151 and FB128 demonstrated that the gut microbiome composition significantly influenced AD classification. Sample FB151, from a 94-year-old female with severe frailty (CFS 7), exhibited clear microbiome dysbiosis, characterized by elevated pro-inflammatory species, absence of the beneficial *Faecalibacterium prausnitzii*, and lower diversity (Shannon index: 2.98), which led to a positive AD classification. In contrast, sample FB128, an 81-year-old male with moderate-to-severe frailty (score 6), maintained a more balanced microbiome with protective *Faecalibacterium prausnitzii* (1.28183) that counterbalanced the presence of AD-associated *Neglecta timonensis*, resulting in higher diversity metrics (Shannon, 3.50; Simpson, 0.93) and a negative classification despite a similar malnutrition risk (score 2). SHAP analyses for both patients highlighted the critical importance of *Faecalibacterium prausnitzii* as a protective factor and *Neglecta timonensis* as a risk-inducing factor, demonstrating how microbiome balance can mitigate clinical risk factors and potentially protect against AD development, even in advanced age and frailty.

**FIGURE 8.**
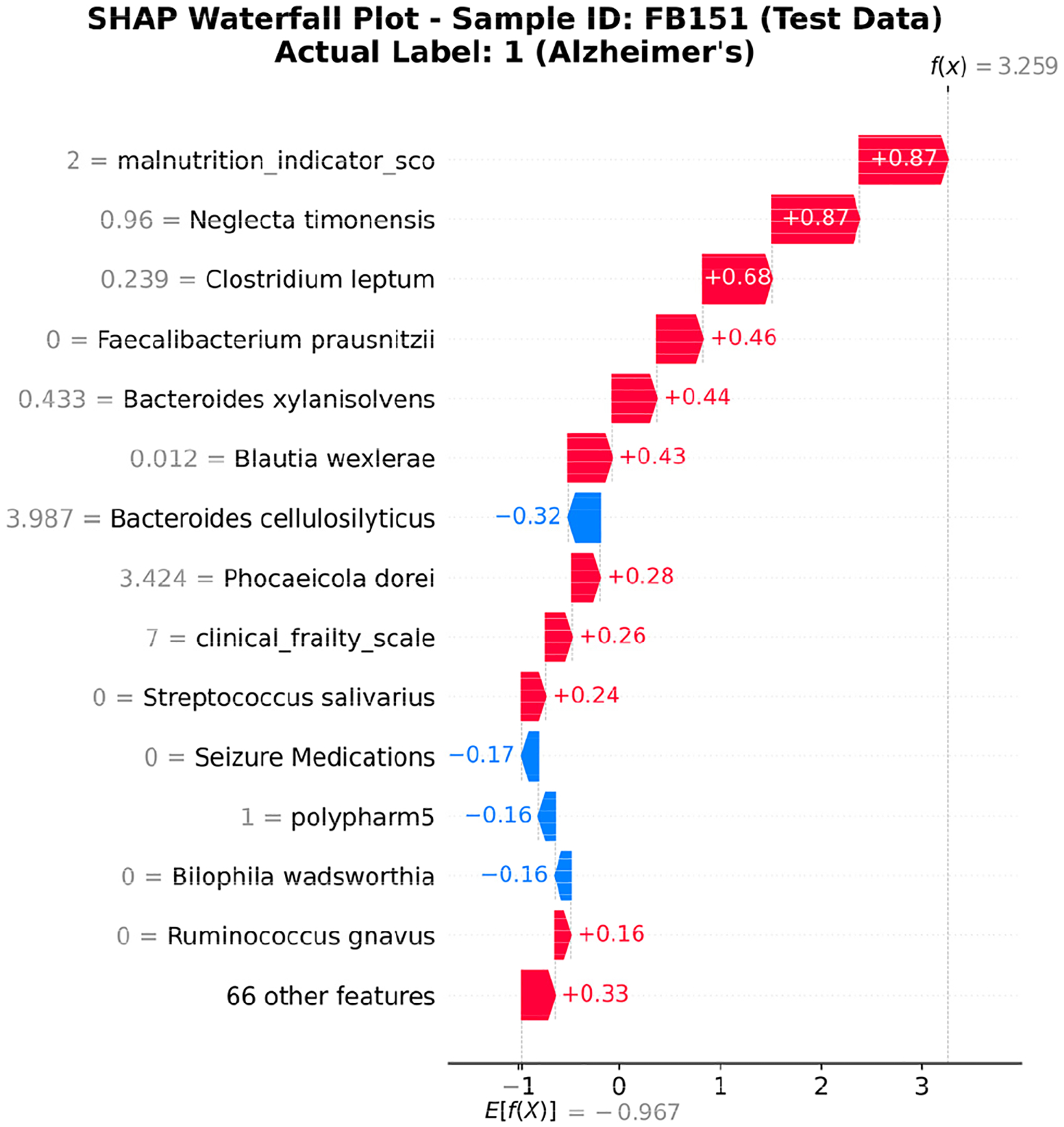
SHAP waterfall plot for Sample FB151 (AD-positive). Malnutrition and *Neglecta timonensis* contributed strongly to the prediction, while the absence of protective *Faecalibacterium prausnitzii* further increased AD risk.

**FIGURE 9.**
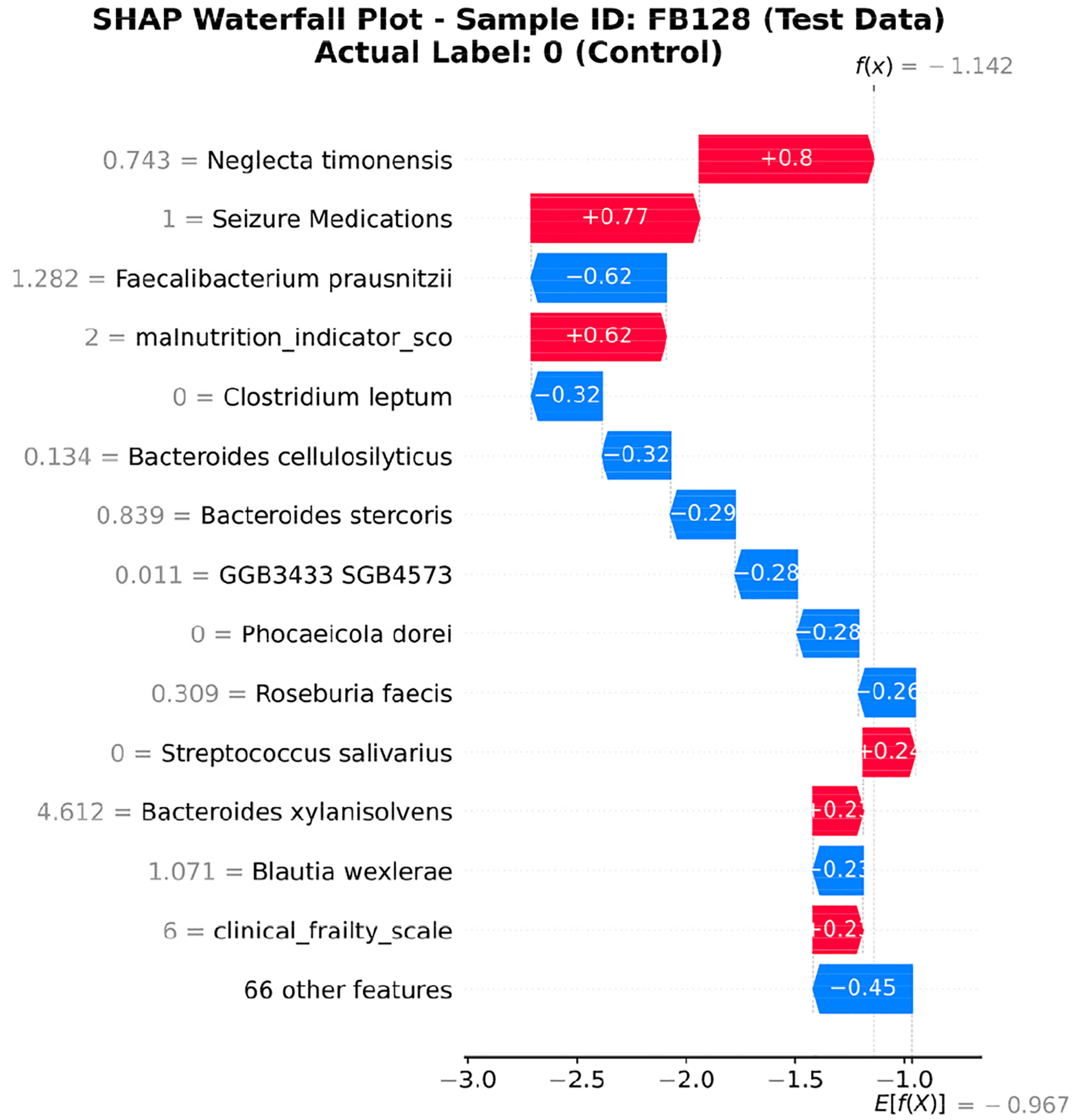
SHAP waterfall plot for Sample FB128 (AD-negative). The presence of *Faecalibacterium prausnitzii* lowered the prediction probability, counteracting other risk factors such as *Neglecta timonensis* and seizure medications.

Figures 8 and 9 show SHAP waterfall plots for the same two samples. These plots display how individual features contributed to the final model prediction and visually support the textual reports in Table 4. In Figure 8, the model gave a high probability of Alzheimer’s disease for FB151, mainly driven by the malnutrition score, presence of *Neglecta timonensis*, and absence of *Faecalibacterium prausnitzii*. These factors increased the prediction toward AD, aligning with the patient’s clinical frailty and gut dysbiosis. In Figure 9, the SHAP values for FB128 demonstrate how protective microbial features, such as *Faecalibacterium prausnitzii*, counteract the effects of seizure medications and *Neglecta timonensis*, reducing the predicted risk and supporting a non-AD classification. The inclusion of these visual explanations clarifies the alignment between computational attributions (SHAP+XGBoost) and the semantic narrative generated by the LLM agent. This combined approach strengthens interpretability and clinical relevance, satisfying both statistical and explanatory transparency.

## IX. DISCUSSION AND LIMITATIONS

The ADAM framework demonstrates the potential of leveraging LLMs via API calls by integrating bioinformatics, machine learning, explainable AI, and relevant literature with biological laboratory data analytics. RAG is facilitated through a web service hosting vector databases. ADAM is tailored to experimental settings common in Alzheimer’s disease research, where around 100 to 300 data points are generated per experiment due to the high cost. We have also successfully run ADAM on low-resource lab computers, such as a Dell Inspiron (AMD Ryzen 5, 8 GB RAM) and an ASUS Vivobook S 14 (Intel Core Ultra 5, 16 GB RAM), where machine learning hardware requirements and GPT API availability bottleneck performance. Large-scale dataset experiments are beyond the scope of the current study. Biological datasets often exhibit substantial noise, which can hinder downstream analyses and affect the robustness of scientific conclusions. When dealing with human-derived data, the levels of noise and inter-individual variability are typically higher, introducing additional complexity to analysis and interpretation. A primary goal of ADAM is to reduce reliance on manual intervention by employing systematic reasoning, guided by literature, data, and computational logic, to identify and mitigate the influence of confounding factors through integrated computational and semantic strategies on a case-by-case basis.

Despite these challenges, ADAM demonstrates that when combined with RAG, CoT reasoning, and traditional machine learning models, large language models can effectively contextualize biological data, even in small-sample or high-noise scenarios such as the data used in this study, which was collected from five nursing home sites. In ADAM, CoT reasoning is guided by RAG to retrieve literature-grounded evidence for each inference step, while traditional models contribute statistical robustness. This combination yields both computational precision and semantically grounded outputs, thereby reducing hallucination and enhancing reliability. This capability is particularly crucial in microbiome clinical research, where important patterns are often subtly dispersed across different data types. Employing explainable AI methods, such as SHAP, enhances interpretability, allowing researchers to connect a model’s predictions to specific features or insights drawn from the literature.

However, this study has some limitations that should be acknowledged. First, although the evaluation was performed on a relatively small but high-dimensional dataset, the ADAM framework was designed to support various types of biological data by using a modular, agent-based architecture. This includes distinct agents for computation, summarization, and classification, each of which can be independently scaled and parallelized to handle increasing data complexity and volume. However, performance at larger scales still needs systematic validation. Second, while the ADAM framework demonstrated strong generalization in this study, additional training, fine-tuning, and reinforcement learning strategies are recommended to reduce hallucinations when applied to unseen datasets or unfamiliar biomedical areas. Third, although the semantic search engine includes a broad literature corpus to reduce noise and variability in raw data and intermediate outputs, its effectiveness depends on the coverage, quality, and representativeness of the embedded knowledge. Gaps or biases in the literature may lead to missed connections or skewed interpretations, notably in underrepresented disease areas. Additionally, the system currently relies on two LLMs pretrained for general-purpose use, which may lack the domain-specific accuracy needed for tasks in biological and clinical settings where small differences can be critical. Finally, the reasoning mechanisms within the summarization and classification agents are manually tuned, which may limit adaptability across different datasets. Future improvements will involve automated tuning and domain-specific optimization to enhance portability and reduce reliance on manual adjustments.

Confounding remains a significant obstacle in modeling Alzheimer’s disease because of clinical heterogeneity. We included all structured clinical and microbiome features in the model to enable data-driven adjustment of observed covariates, using XGBoost with regularization and embedded feature selection. Known confounders such as polypharmacy and proton pump inhibitor use appeared as top contributors. However, causal inference methods were not used, and residual confounding may still be present. SHAP values help interpretability but do not establish causality, so findings should be viewed with caution.

In addition, while ADAM can operate on modest hardware by leveraging GPT API calls, the computational overhead of locally running multiple AI agents and performing real-time retrieval over large document bases may limit practicality in laboratory settings with constrained infrastructure. Furthermore, although ADAM enables comprehensive data analysis and biological reasoning, expert interpretation is necessary to validate the findings and ensure scientific rigor. It is also important to recognize that the large language models used in this study have inherent limitations in consistency, as testretest reliability remains imperfect even when the generation temperature is set to zero [48], [49]. Given the biological nature of the data and non-deterministic behavior of these models, we repeated result generation 30 times, following the Central Limit Theorem, to support the statistical reliability of our conclusions. The findings are further supported and contextualized using the most relevant content retrieved from a corpus of 76,751 peer-reviewed publications on Alzheimer’s disease, which are integrated into the system’s semantic retrieval engine.

## X. CONCLUSION AND FUTURE WORK

This study offers a thorough evaluation of the ADAM framework for classifying AD by comparing its performance with that of the widely used machine-learning baseline, XGBoost. By leveraging LLMs, explainable AI, and retrieval-augmented generation, ADAM achieved better predictive performance and showed greater consistency across repeated experimental runs.

Across 30 independent trials, ADAM consistently achieved a higher mean F1 score (0.7263) than XGBoost (0.6774), with a statistically significant difference confirmed by the Mann-Whitney U test (p = 0.0418). The lower standard deviation of the ADAM F1 scores (0.0632 vs. 0.1217) and its favorable distribution characteristics further underscore its stability and robustness. The F1 variance of XGBoost (0.0148) indicated that XGBoost shows 3.71 times more variability than ADAM (0.0040), as supported by Levene’s test (p = 0.0300). These performance characteristics are particularly important in the medical domain, where model stability and reproducibility are essential for building trust in diagnostic tools and supporting consistent decision-making in patient care.

The ADAM framework offers a promising direction for integrating AI-driven inference with biological and clinical data, providing higher accuracy, greater interpretability, and operational stability. These results support its potential for adoption in biomedical research environments and clinical decision support systems, particularly in settings characterized by small, noisy, or imbalanced datasets. Future work will examine scalability and generalizability across more complex, multimodal biological inputs, including integrated multi-omics datasets, to broaden the framework’s applicability and performance. It will also examine the integration of causal inference methods to enhance confounder adjustment and improve the interpretability of feature-outcome relationships in high-dimensional observational data.

Overall, ADAM addresses the crucial gap between experimental biology and agentic AI. Future enhancements, such as domain-specific LLMs and fine-tuning, expanded knowledge integration with RAG model optimization, adaptive reasoning logic using reinforcement learning, and large-scale, real-time validation mechanisms, are essential for broader adoption and translational impact. The reasoning logic will include three modes: manual, automatic, and hybrid, in a future release of ADAM, allowing flexible adaptation across various research and data environments. These developments may ultimately enable a foundational LLM trained and explicitly tuned for AD research, driving this line of research toward the realization of physical AI systems capable of interacting with and reasoning about complex biological data in real-time. It is important to note that ADAM is currently designed as a research tool only and should not be used for clinical application in its current form. This represents a promising future development for ADAM, which, with further enhancement and validation, could be adapted to other datasets and clinical settings while offering clearer, more user-friendly interpretation of its outputs.

## Figures and Tables

**TABLE 1.** Publications and text segment counts by topic.

Keywords	Publications	Segments
Alzheimer’s disease	62,478	1,591,441
Gut Microbiome	11,692	381,630
Immunosenescence	1,273	36,172
Gut-Brain Axis	1,308	49,259
Total	76,751	2,058,502

The number of publications and segmented text units analyzed in this study across key biomedical topics related to Alzheimer’s disease and microbiome research. The dataset includes over 76,000 publications and 2 million segments, MOST focused on Alzheimer’s disease and the gut microbiome.

**TABLE 2.** Embedding Model Comparison. Summary of key performance and usability attributes of the ADA-002 embedding model, highlighting its strong semantic representation, high computational efficiency, moderate storage demands, and broad adoption across applications.

Criterion	ada-002 (1,536 dims)	3-small (1,536 dims)	3-large (3,072 dims)
Semantic Richness	High	High	Very High
Computational Efficiency	High	Highest	Moderate to Low
Storage Requirements	Moderate	Moderate	High
Cost-effectiveness	High	Moderate	Lower
Versatility & Adoption	Proven, widely used	Newer, less proven	Newer, powerful, but resource-intensive

**TABLE 3.** Base model performance averaged across 10 random seeds.

Model	Accuracy	AUC	F1	Mean
XGBoost	**0.769 ± 0.069**	**0.821 ± 0.061**	**0.651 ± 0.1**	**0.769 ± 0.069**
Random Forest	0.742 ± 0.066	0.804 ± 0.087	0.603 ± 0.087	0.742 ± 0.066
Logistic Regression	0.735 ± 0.067	0.772 ± 0.1	0.626 ± 0.095	0.735 ± 0.067

The classification performance of XGBoost, Random Forest, and Logistic Regression models was evaluated using mean accuracy, AUC, and F1 scores with standard deviation. Results reflect model stability and predictive power across repeated runs, with XGBoost achieving the highest average performance across all metrics.

**TABLE 4.** ADAM Sample Reports: Positive Classification and Negative Classification. Interpretability-focused summaries produced by ADAM for one positively and one negatively classified sample. Each report includes clinical variables, medication usage, gut microbiome composition, diversity metrics, and SHAP-based feature attributions to clarify the predicted Alzheimer’s disease status.

Sample of a Positive Classification - Conclusion **Prediction: Yes** - The prediction of Alzheimer’s disease (AD) for Sample ID FB151 is strongly supported by multiple factors:	Sample of a Negative Classification - Conclusion **Prediction: No** - The prediction for Sample ID FB128 is ‘No’ for Alzheimer’s disease (AD) based on a probability of 24.20%. This low probability is influenced by several key factors:
1. **Clinical Indicators:** The patient is a 94-year-old female with a Clinical Frailty Scale score of 7, indicating severe frailty. This level of frailty is significantly associated with cognitive decline and AD progression. Additionally, the patient has a malnutrition score of 2, which places her at risk for nutritional deficiencies that can exacerbate cognitive decline.	1. **Demographics and Clinical Background:** - The patient is 81 years old, male, with a moderate to severe frailty score of 6. This level of frailty is concerning but does not alone indicate a high probability of AD. – The malnutrition score of 2 indicates a risk of malnutrition, which can exacerbate cognitive decline but is not definitive for AD.
2. **Polypharmacy:** The patient is on multiple medications (polypharmacy), which can alter gut microbiota composition and increase systemic inflammation, further contributing to cognitive health deterioration.	2. **Clinical Features:** - The presence of seizure medications and oral corticosteroids suggests potential neurological comorbidities, which could elevate AD risk. However, the absence of cardiovascular risk factors (e.g., hypertension, high cholesterol) is protective against vascular contributions to cognitive decline.
3. **Gut Microbiome Profile:** The microbiome analysis reveals an imbalance with elevated levels of pro-inflammatory species such as *Phocaeicola vulgatus* and *Clostridia bacterium*, while beneficial species like *Faecalibacterium prausnitzii* are absent. This dysbiosis is linked to increased inflammation and cognitive decline, which are critical factors in AD pathology.	3. **Gut Microbiome Profile:**- The microbiome analysis shows a moderate abundance of protective species like *Faecalibacterium prausnitzii* (1.28183), which has anti-inflammatory properties. This is counterbalanced by the presence of *Neglecta timonensis* (0.74327), which is positively associated with AD risk. – The overall alpha diversity metrics (Shannon Index: 3.50, Simpson Index: 0.93) indicate a relatively balanced microbiome, which is generally protective against systemic inflammation and cognitive decline.
4. **Diversity Metrics:**- The Shannon Index of 2.98 indicates moderate diversity, but the presence of dominant pro-inflammatory species and high Bray-Curtis dissimilarity suggests significant deviations from a healthy microbiome. This imbalance can exacerbate systemic inflammation and cognitive decline.	4. **SHAP Feature Importance:**- The top SHAP features include *Neglecta timonensis* (SHAP: +0.7978), Seizure Medications (SHAP: +0.7696), and *Faecalibacterium prausnitzii* (SHAP: −0.6193). The positive contributions from Neglecta and seizure medications suggest increased risk, while Faecalibacterium’s negative contribution indicates a protective effect.
5. **SHAP Feature Importance:** The SHAP analysis highlights that the malnutrition score, the presence of *Neglecta timonensis*, and the low levels of *Faecalibacterium prausnitzii* are the top contributors to the high probability of AD. These features align with known risk factors for AD.	5. **Diversity Metrics:** - The Bray-Curtis dissimilarity indicates high dissimilarity from healthy controls, suggesting a distinct microbial composition that may reflect gut dysbiosis linked to AD.

## Data Availability

All data are available under BioProject accession number PRJNA529586 at NCBI and are further described in the Supplementary Data at doi.org/10.1128/mBio.00632-19 at mBio.

## CODE AVAILABILITY

The complete code that supports all the findings and analyses presented in this study is available at https://github.com/melhzy/ADAM. This repository includes data preprocessing scripts, model training scripts, evaluation scripts, and documentation detailing the setup procedures and dependencies. The code is distributed under the MIT License, permitting reuse and modification with appropriate attribution. Please use the repository issue tracker or contact the corresponding author, Dr. Ziyuan Huang, for any questions or concerns. For inquiries related to Alzheimer’s and its microbiome, please contact Dr. John P. Haran. Dr. Bucci Vanni should be contacted for computational biology inquiries.
